# Chitin Increases *Mycobacterium ulcerans* Growth in Acidic Environments

**DOI:** 10.1264/jsme2.ME17160

**Published:** 2018-06-16

**Authors:** Daniel Sanhueza, Christine Chevillon, Nicolas Bouzinbi, Sylvain Godreuil, Jean-François Guégan

**Affiliations:** 1 MIVEGEC, IRD, CNRS, Université de Montpellier Montpellier France; 2 Laboratoire de bactériologie, CHU de Montpellier, Université de Montpellier France; 3 International United Nations Program FutureEarth, OneHealth Core Research Program Montreal Canada

**Keywords:** *Mycobacterium ulcerans*, environmental mycobacteria, experimental test, ecological niche, neglected tropical disease

## Abstract

Species with a chitinous exoskeleton are overrepresented among the aquatic organisms carrying *Mycobacterium ulcerans* (MU) in nature and laboratory experiments have demonstrated the enhancing effects of chitin on the growth of MU. Field surveys identified pH as one of the key parameters delineating the distribution of MU in tropical regions. The present study investigated the relationship between chitin and pH in MU growth. By focusing on pH variations in the field, our results revealed that chitin enhanced MU growth in acidic environments. The present study provides new information on the ecological conditions favoring the development of this mycobacterium in nature.

*Mycobacterium ulcerans* (MU) is the etiological agent of Buruli ulcer (BU), a neglected human infectious disease reported in more than 30 tropical and subtropical countries that is responsible for skin ulcers and/or disabilities in the absence of treatment (1). This mycobacterium has been associated with a large diversity of aquatic host taxa (2, 7, 8, 13, 16, 23–25), and recent field observations identified several environmental conditions that appear to be associated with a shift in the distribution of MU in tropical freshwater ecosystems. These key conditions involve not only the composition and diversity of the assemblages of aquatic species that are locally present (9, 10, 12, 21), but also physicochemical properties, such as the water flow speed, quantity of dissolved oxygen, and pH (8, 9, 11, 18). If field data remain scarce, it is important to note that the overlaps in pH values recorded across MU-positive sites from different tropical regions (8, 15) range between 4.5 and 7.5 (Supplementary file 1). This finding is consistent with laboratory research showing MU optimal growth *in vitro* for pH values ranging between 5.4 and 7.2 (19).

Since chitin is a major component of the cuticle of arthropods and arthropods are overrepresented in MU-associated taxa in BU endemic areas (9, 14, 16), the hypothesis that chitin may directly play a role in the development of MU was proposed. An experimental test recently confirmed this hypothesis and the availability of chitin was found to promote MU growth *in vitro* more strongly than other modifications in the concentrations of inorganic or organic elements commonly observed in the field (22).

The present study attempted to provide a clearer understanding of the biology of MU by (i) using pH variations recorded in BU endemic areas to investigate the impact of pH modifications on MU growth under controlled laboratory conditions (Supplementary file 1), and (ii) testing whether the impact of pH on MU growth is associated with the presence/absence of chitin in culture medium.

The present study did not involve any human or animal participants, only an *in vitro* culture of the 1G897 laboratory strain isolated from human biopsies in 1991 (6). We cultured the 1G897 strain at 30°C in tubes containing 27 mL of 7H9 medium and 3 mL of the growth supplement OADC (Oleic, Albumin, Dextrose, and Catalase) (Sigma-Aldrich, St Louis, MO, USA). This standard medium culture defines 6.7 as the control pH value. We also used seven experimental pH values (4.5, 5.0, 5.5, 6.0, 6.5, 7.0, and 7.5) in order to cover the field variations observed in BU endemic areas (9, 16) (Supplementary file 1). These experimental pH values were obtained by adding a 1:10 v/v dilution of acid phosphoric solution or sodium hydroxide to 7H9 growth medium where appropriate. Under each pH condition, we tested the effects of the presence of chitin by starting experiments with 10 tubes per pH condition: 5 replicates without chitin and 5 replicates with chitin-supplemented culture medium at a final chitin concentration of 0.5 g 100 mL^−1^. Commercial alpha chitin extracted from shrimp shells (Sigma-Aldrich) was used in the present study.

We monitored pH and the abundance of MU at the launching date (*t*=0) and at *t*=25, 35, and 50 d post-inoculation. In order to modify the experimental volume as little as possible and avoid any pH changes caused by measurements, pH values were recorded with a micro-electrode (Thermo Scientific) adapted to small volumes and by only collecting 500 μL at each measurement. No changes in pH values were observed in any tubes throughout the experiment. DNA was extracted using 100 μL of each bacterium culture by employing a DNeasy Blood & Tissue Kit (Qiagen, Hilden, Germany) according to the “bacteria protocol” recommended by the manufacturer. MU quantification was based on the Real-Time PCR protocols described previously (22). The accuracy of these protocols was tested with external standard curves obtained from serial dilutions of MU (strain 1G897) DNA over five logs (from 10^6^ to 10^2^ units mL^−1^). This allowed the mean inoculums added to each experimental tube at the launching date of the experiment (*t*=0) to be estimated as ~3,000 MU cells per tube.

We defined the estimated number of MU cells per tube as the response variable and analyzed its variation using general linear mixed model (GLMM) procedures (5) in which replicates were fit as random factors, while pH values, chitin concentrations, and time were fixed factors. The maximal model, including all interactions among fixed factors, was simplified by sequentially eliminating non-significant terms, starting by the highest order of interactions among factors, to finally establish a minimal model (5). The significance of each term was established using either a likelihood ratio (*LLR*) test, which is approximately distributed as a χ^2^ distribution (4), or an *F* test (5). Analyses and graphics were performed with the *R* statistical software, version 3.3.0 (20) and the *R* packages *MASS*, *nlme*, *lme4*, *beeswarm*, *RColorBrewer*, *and ggplot2*, or with the software GraphPad prism 5, version 5.02.

The experimental set-up confirmed that MU exhibited the ability to survive and grow over all the pH ranges observed in BU endemic regions, with the extremes (pH=4.5 or 7.5) being less suitable for its development *in vitro* (Fig. 1). The minimal GLMM evidenced independently significant effects of the factors ‘Time’ (*F* value=13.261, *P* value<0.0001) and ‘Chitin’ concentrations (*F* value=4.679, *P* value=0.0379) as well as of the interaction between ‘pH’ and ‘Time’ (*F* value= 1.845, *P* value=0.0211) or ‘Chitin’ and ‘Time’ (*F* value= 9.861, *P* value=<0.0001; Supplementary file 2). In contrast, the interaction between ‘pH’ and ‘Chitin’ concentrations was not significant (*F* value=1.980, *P* value=0.088; Supplementary file 2). Nevertheless, MU grew faster in chitin-supplemented media with pH≤6.5 than in chitin-free media with 6.7≤pH≤ 7.5; if the maximal abundance of MU was similar across these experimental conditions (*F* value=3.587, *P* value=0.22), the maximum was reached within 25 to 35 d in chitin-supplemented media with pH≤6.5, but only in 50 d in chitin-free media with pH≥6.7 (Fig. 1).

In order to clarify the relationship between chitin concentrations and pH conditions, we pooled pH experimental conditions into three classes: ‘low’ regroups the three lowest experimental values (4.5, 5.0, and 5.5), ‘middle’ regroups the two intermediate values (6.0 and 6.5), and ‘high’ joins the two remaining experimental values (7.0 and 7.5). This led to the same results as those obtained when considering experimental pH values individually (*LLR*=33.202, *P* value=0.13). The effects of ‘Chitin’ concentrations on variations in the estimates of bacterial cells across replicates and experimental conditions remained significant in the low and middle groups (*i.e.*, for 4.5≤pH≤6.5; *F* value=4.394, *P* value=0.0055; Supplementary Material 3), but not in the high group (*i.e.*, for pH>6.5; *F* value=2.758, *P* value=0.0747). Interestingly, this may be correlated with other differences among experimental conditions: MU kinetic growth stopped after 25 to 35 d in chitin-supplemented media for pH values in the low or middle groups, but otherwise remained constant throughout the 50 d of monitoring (Fig. 2).

Several laboratory experiments and/or field surveys have attempted to elucidate the impact of environmental variables on MU growth *in vitro* or its presence in natural ecosystems. However, the present study was the first to experimentally investigate the impact of pH field variations reported in BU endemic areas on MU growth *in vitro*. Since the addition of chitin to standard culture medium was previously reported to stimulate MU growth *in vitro* at pH=6.7 (22), the present study attempted to elucidate the mechanisms by which the growth stimulating effects of chitin interplay with the effects on MU growth caused by variations in pH.

The present results on the individual effects of pH values are consistent with previous findings on the optimal conditions for *in vitro* MU cultivation (19). The optimal conditions for *in vitro* MU cultivation correspond to the present ‘control’ conditions under which the MU doubling time was 3.5 d (15). The difference in MU growth between optimal pH (6.7 in the control) and the experimental value of 6.5 may be attributed to H_3_PO_4_ being added to standard culture medium in order to reach more acidic experimental values. Some bacteria are known to use PO_4_^−3^ as a phosphate source, which is essential for the biosynthesis of nucleic acids (12); however, this has not yet been examined in MU. It is important to note that we previously demonstrated that the concentration of PO_4_^−3^ did not significantly affect MU growth (22).

The present results revealed the pH-mediated effects of chitin on MU growth, namely, the enhanced development of MU cells in acidic environments in the presence of chitin. Based on the presence of three genes coding for chitinases (MUL_2681, MUL_2210, MUL_0371) in the MU genome, one likely explanation for these complex MU kinetics in chitin-supplemented culture media is a decrease over time in the availability of chitin. Alternatively, since growth media was not renewed throughout the experiment, MU growth may have stopped after ~25 to 35 d in chitin-supplemented media because of the accumulation of sub-products resulting from the MU metabolic use of chitin in the early stages of experimentation. Further research, including the monitoring of chitin availability during MU growth, appears to be necessary in order to clarify the exact mechanisms underlying the relationship among chitin, pH, and MU, as revealed in the present study.

Because we have found no statistical effect of this solution on MU growth, it seems plausible that acid phosphoric is not used by MU as a phosphate source, thus it may affect MU growth indirectly e.g. by changing the available concentrations and compositions of chitin-derived nutrients in culture medium. Although this study did not confirm this hypothesis in a culture media with and without H_3_PO_4_, further investigations will clarify the effects of PO_4_^−3^ on the physical and chemical states of chitin in media.

If the initial effects of chitin supplementation on MU growth is clarified (*e.g.*, fixation support, nutrients used for MU metabolism, or both) (22), it is noteworthy that MU is not the only environmental bacterium sensitive to the presence of chitin. The presence of this polysaccharide accounts for approximately 40% of the variations observed in the abundance of *Vibrio cholerae* in natural environments (17). Moreover, longitudinal field surveys performed in BU endemic areas already demonstrated the co-occurrence of temporal seasonality (between dry and rainy seasons) and spatial variations in pH and the abundance of MU with fluctuations in the abundance of aquatic arthropods, and, thus, in chitin availability (8, 9, 14).

The present results, which were obtained from a rather simple experimental design, provide some explanations from these complex field observations: seasonal variations in pH and species assemblages within aquatic ecosystems may account for most of the seasonal variations in MU abundance, as exemplified by the relationship between low pH values and chitin availability. Furthermore, chitin is known for its antimicrobial effects against many MU competitors, including bacteria, yeast, and fungi (3). Since many of these competitors grow slightly faster than MU, the stimulating effects of chitin on the growth of MU at low pH (*i.e.*, under conditions that are not suitable for the growth of MU in the absence of chitin, Fig. 1) combined with the adverse impact of chitin on many MU competitors suggest that the presence of chitin in nature may increase its growth rate, thereby indirectly increasing the competitive ability of MU towards co-existing microorganisms under these environmental conditions.

To conclude, the present study elucidated the roles of pH and chitin in MU cell growth *in vitro* and also demonstrated for the first time a strong relationship between chitin and pH, indicating that the enhancing effects of chitin on the growth of MU are the strongest in more acidic environments. This study provides new information on the multifactorial process playing a role in the development of MU, and gives a novel insight into the ecological niche of this environmentally persistent mycobacterium.

## Supplementary Material



## Figures and Tables

**Fig. 1:**
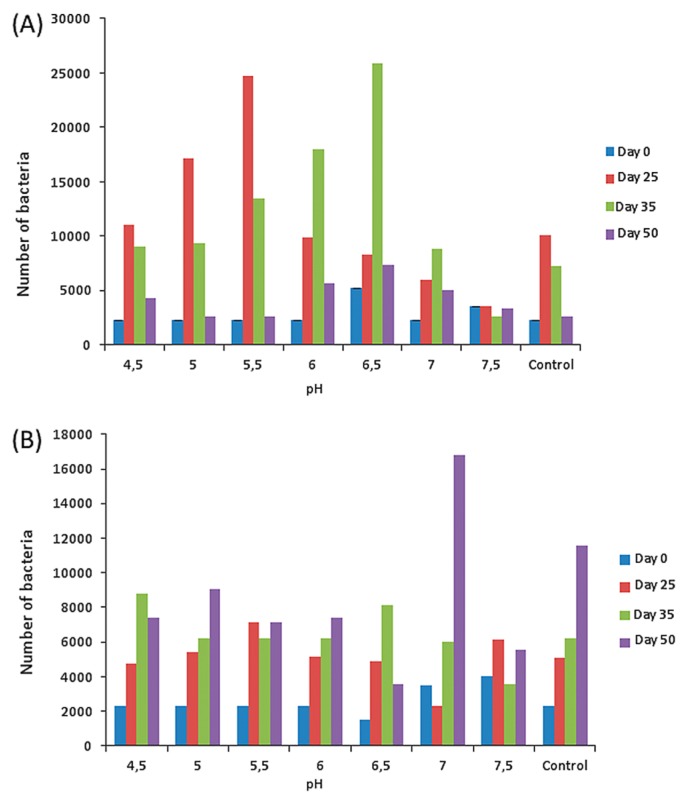
Mean estimates in numbers of bacterial cells across pH conditions in the presence (panel A) or absence (panel B) of chitin. The experiment started on day 0; pH=6.7 in the control.

**Fig. 2:**
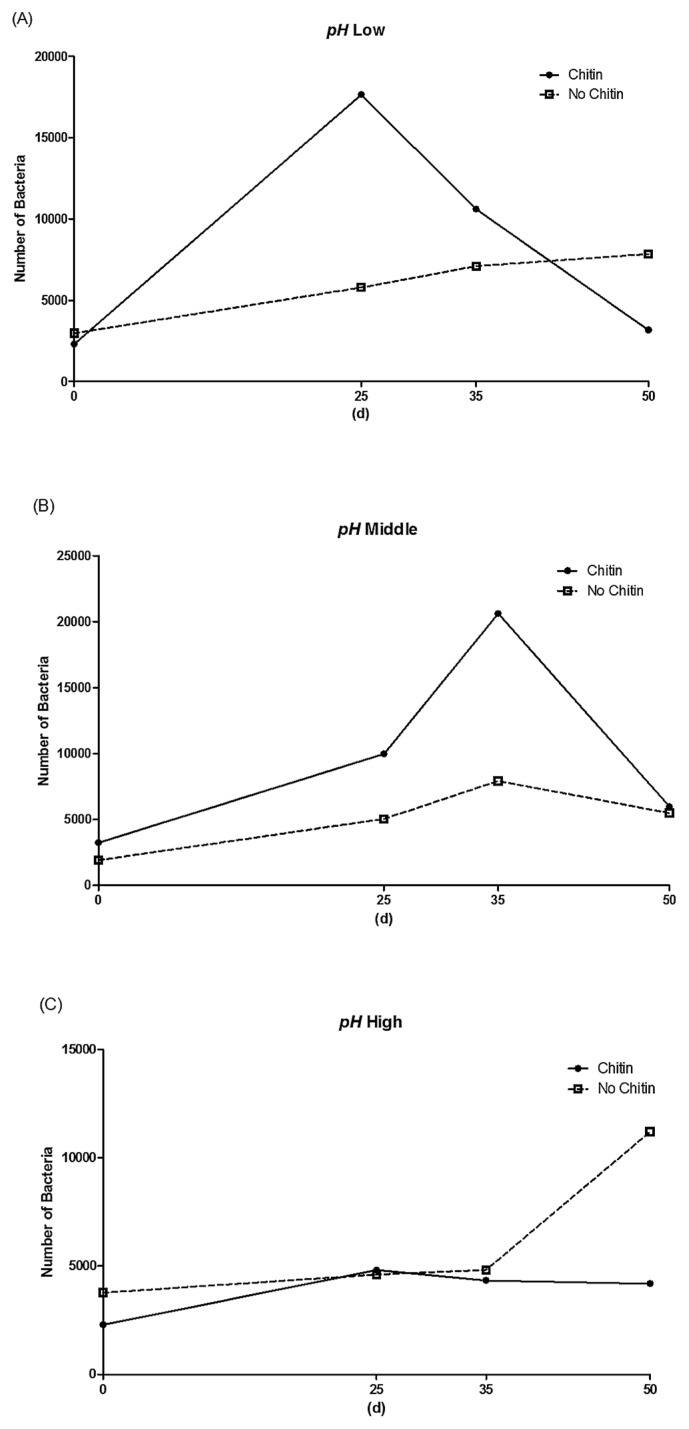
Interactions between pH conditions and the presence/absence of chitin on MU kinetic growth. pH values were pooled into three groups for clarity: (A) low (4.5≤pH≤5.5), (B) medium (6.0≤pH≤6.5), and (C) high (7.0≤pH≤7.5).
