# Supplementary material for: Chitin Increases Mycobacterium ulcerans Growth in Acidic Environments
Source: Microbes Environ. 2018 Jun 16;33(2):234–7. doi: 10.1264/jsme2.ME17160 (PMC6031397; doi:10.1264/jsme2.ME17160)

Supplementary Material SM1-to-SM3

SM1 . **Distributions in the *pH*-values among aquatic environments.** Distributions are given for three BU endemic areas: (A) Akonolinga, Cameroon; (B) Bankim, Cameroon and (C) French Guiana. (From the data published in Garchitorena *et al.* 2015a and Morris *et al.* 2014, 2016). Dotted lines delineate the range of *pH* values used in our experiments. The black arrow indicates the *pH* value of control medium 7H9.

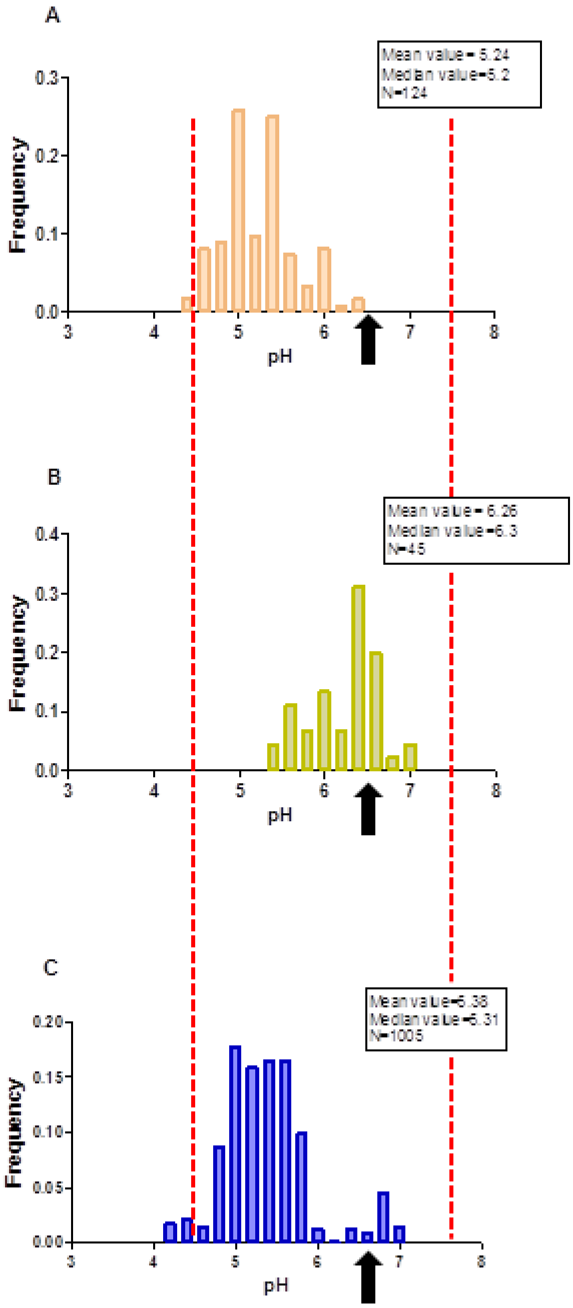

SM 2. **Statistics driven from the maximal (A) and minimal (B) linear mixed models used to fit the number of *Mycobacterium ulcerans* cells.** Models are transcribed in *R* language. In both cases, samples were declared as explanatory variables with random effects. Statistics in bold characters are those that are significant at 5% C.I.

(A) **Maximal model:**

lme (number of bacteria ~ *pH*×Chitin×Time+ *pH*×Chitin +*pH*×Time+ Chitin×Time + Chitin + Time + *pH* , random=~1|Sample,data=data,method="ML)

Explanatory variable	F- value	*p*-value
Time	13.077	**<0.001**
pH	1.632	0.161
Chitin	4.305	**0.046**
pH×Chitin	1.980	0.088
pH×Time	1.820	**0.026**
Chitin×Time	9.724	**<0.001**
pH×Chitin×Time	0.899	0.593

(B) **Minimal model:**

lme(Numberbacteria~ *pH*×Chitin +*pH*×Time+ Chitin×Time + Chitin + Time + *pH* ,random=~1|Sample,data=data,method="ML")

Explanatory variable	F- value	p-value
Time	13.261	**<0.001**
pH	1.610	0.167
Chitin	4.679	**0.038**
pH×Chitin	2.008	0.084
pH×Time	1.845	**0.021**
Chitin×Time	9.861	**<0.001**

SM 3. Changes in the number of *Mycobacterium ulcerans* cells within three *pH* groups. The three *pH* groups are defined as follows: *pH* low (4.5, 5.0, 5.5); *pH* middle (6.0, 6.5) and *pH* high (7.0, 7.5). Each color line represents a different time (*t* = 0, 25, 35 and 50).

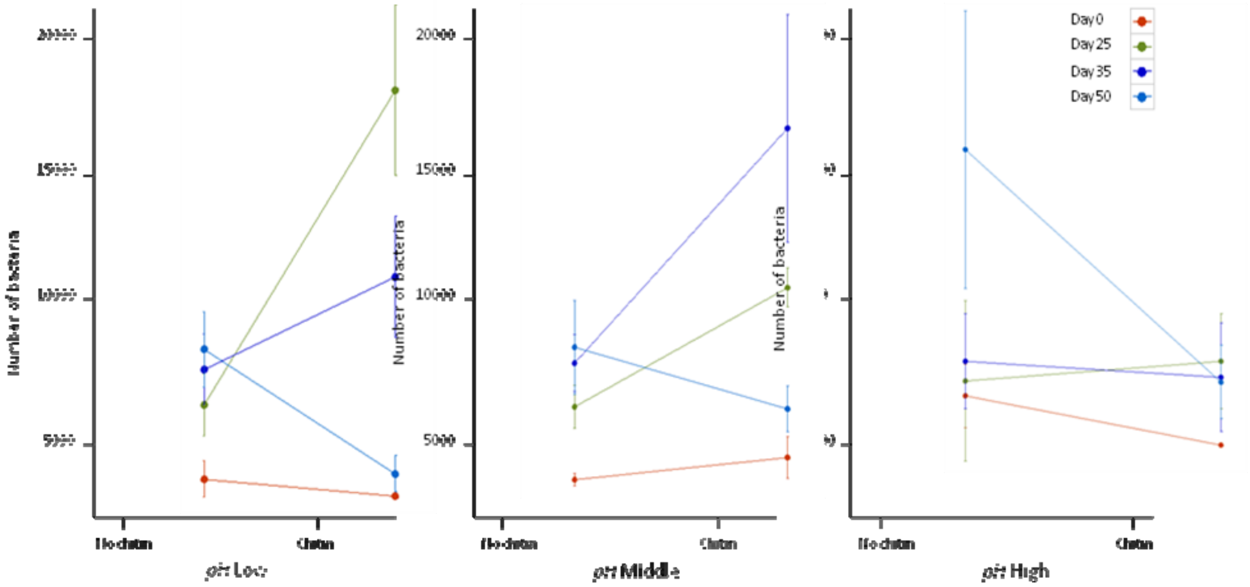

Supplement: Supplementary file 1 [file 33_234_s1.pdf]
